# A Rare Complication in the Delayed Manifestation of Bochdalek Hernia During Pregnancy: A Case Report

**DOI:** 10.7759/cureus.40718

**Published:** 2023-06-21

**Authors:** Aneena A Moncy, Ashok Ninan Oommen, Rejana R Joy

**Affiliations:** 1 General Surgery, Jubilee Mission Medical College and Research Institute, Thrissur, IND

**Keywords:** left bochdalek hernia, gastric outlet obstruction, diaphragmatic hernia pregnancy, adult congenital diaphragmatic hernia, organo-axial gastric volvulus

## Abstract

Acute gastric volvulus is a surgical emergency and is known to occur secondary to diaphragmatic hernia and eventration. Adult presentation of congenital diaphragmatic hernia is rare, with an estimated incidence of around 0.17%, and pregnancy may predispose to the development of symptoms due to increased intra-abdominal pressure. Pregnancy complicated by diaphragmatic hernia is associated with a high risk of maternal and fetal mortality, necessitating timely diagnosis and treatment.

We present the case of a 23-year-old female presenting with a symptomatic left Bochdalek hernia complicated by organo-axial gastric volvulus during her second trimester (27 weeks). Emergency laparotomy was performed, with Caesarean section, reduction of gastric volvulus, and mesh repair of the left posterolateral defect.

## Introduction

Late presentation of congenital diaphragmatic hernia (CDH) is rare in adulthood, with a reported frequency of 0.17% to 6% in the literature [1,2]. Common symptoms of adult Bochdalek hernia typically include recurrent abdominal pain (62%), as well as respiratory symptoms (40%) such as chronic dyspnea, chest pain, postprandial fullness, and vomiting. Life-threatening gastrointestinal and respiratory complications can develop when herniated organs undergo obstruction and strangulation [3]. Secondary gastric volvulus has been reported as a rare presentation of Bochdalek hernia [4,5].

The onset of symptoms in previously asymptomatic women with congenital Bochdalek hernia can occur during pregnancy, with 43 cases reported in the literature between 1941 and 2020 [6,7]. The associated difficulty and delay in diagnosis pose a major threat to maternal and fetal well-being. We present the case of a 23-year-old primigravida presenting with a symptomatic left-sided Bochdalek hernia complicated by gastric volvulus in her second trimester.

## Case presentation

A 23-year-old primigravida at 27 + 6 weeks gestation, who has been receiving regular antenatal follow-up, presented with a history of acute upper abdominal pain and multiple episodes of nonbilious vomiting for one day. She had been diagnosed with incidentally detected left-sided diaphragmatic eventration eight years prior and was not advised surgical intervention as she was asymptomatic. Regular antenatal sonological evaluation had reported elevated left hemidiaphragm with no evident visceral herniation and normal fetal development; hence, the patient had been advised expectant management till term.

**Figure 1:**
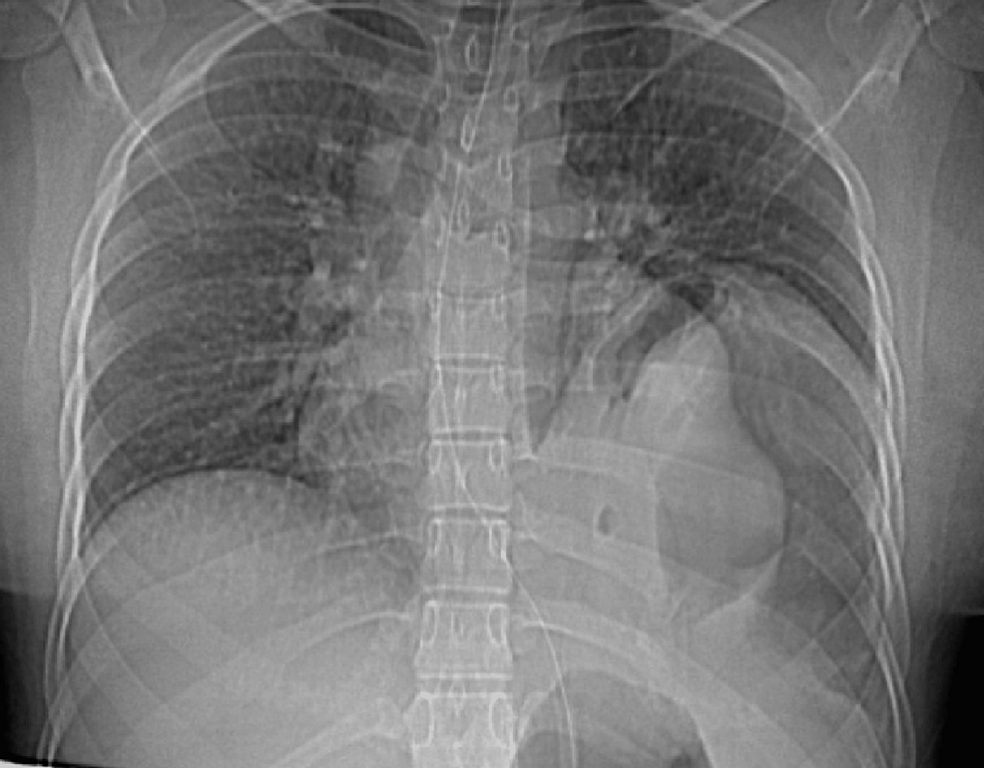
Chest radiograph AP view at presentation, after the passage of the nasogastric tube, showing the elevated left hemidiaphragm and gastric shadow. AP, anteroposterior

At presentation, she was tachycardic, with a soft gravid abdomen on examination and reduced breath sounds over the left thorax. The initial ultrasound showed that the stomach and left kidney were located in the left lower thorax, indicating a significant gastric outlet obstruction. Additionally, the ultrasound confirmed good fetal cardiac activity. Nasogastric aspiration was initiated and an erect chest X-ray was taken with abdominal shielding (Figure 1), which showed an elevated left hemidiaphragm. Serum lipase was found to be elevated at 1,166 U/L.

**Figure 2:**
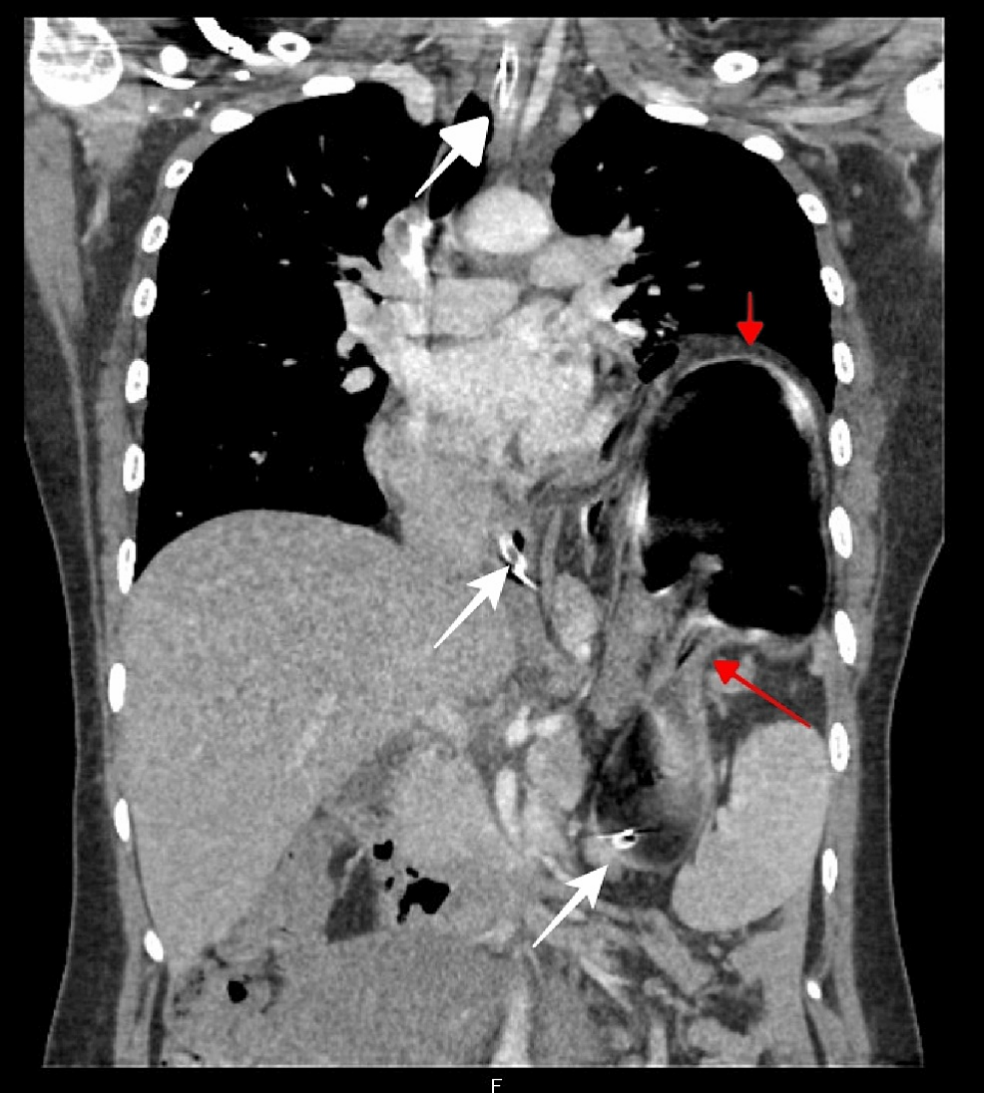
CT thorax and upper abdomen coronal section showing diaphragmatic herniation of stomach with organo-axial volvulus (red arrows) and a nasogastric tube inserted (white arrows). CT, computed tomography

**Figure 3:**
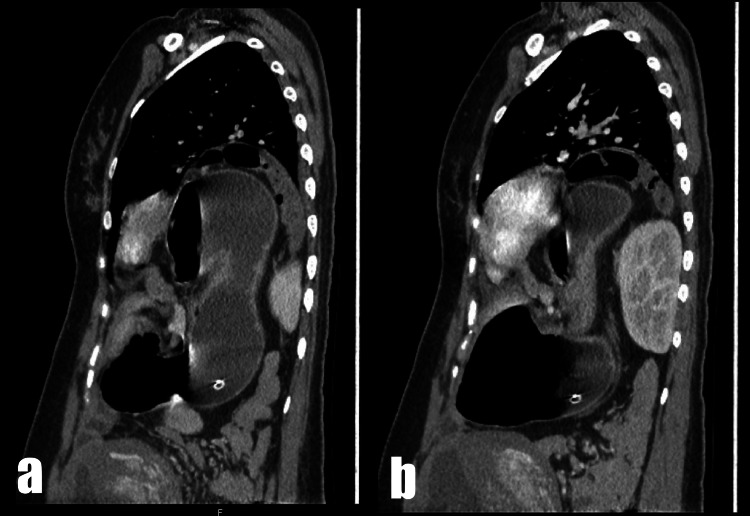
CT thorax and upper abdomen sagittal sections showing (a) posterolateral diaphragmatic herniation of stomach and (b) ectopic left kidney. CT, computed tomography

**Figure 4:**
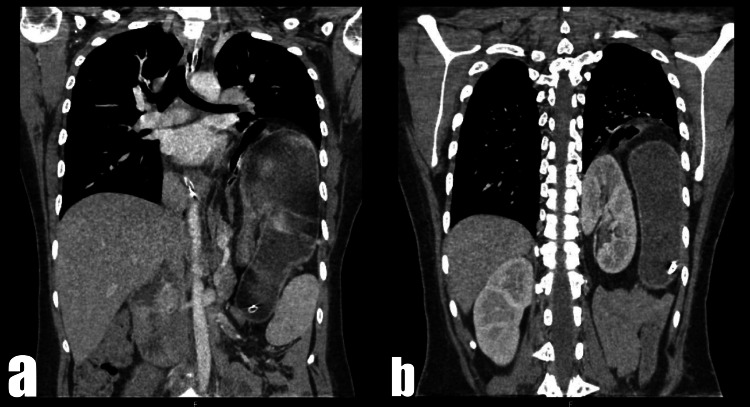
CT thorax and upper abdomen coronal sections showing (a) herniation of the stomach and (b) ectopic location of left kidney. CT, computed tomography

Despite fluid and electrolyte resuscitative efforts, the patient developed respiratory distress with severe respiratory and metabolic alkalosis (pH 7.8; pCO_2 _23.7 mmHg; lactate 0.2 mmol/L; HCO_3 _41 mmol/L). Emergency contrast-enhanced computed tomography (CECT) imaging was performed, which showed a left posterolateral diaphragmatic defect measuring 10.2 cm × 9.7 cm. The scan showed herniation of the colon, stomach, spleen, and pancreas and an organo-axial gastric volvulus, resulting in outlet obstruction, and an ectopic left kidney (Figures 2-4).

Due to the maternal respiratory compromise and fetal distress, the managing team, in consultation with the patient's spouse and relatives, decided to proceed with an emergency lower segment Caesarean section (LSCS). The decision took into account the potential risks associated with maternal mortality, premature birth, and fetal/neonatal loss. Neonatology was consulted, and antenatal corticosteroids were administered.

**Figure 5:**
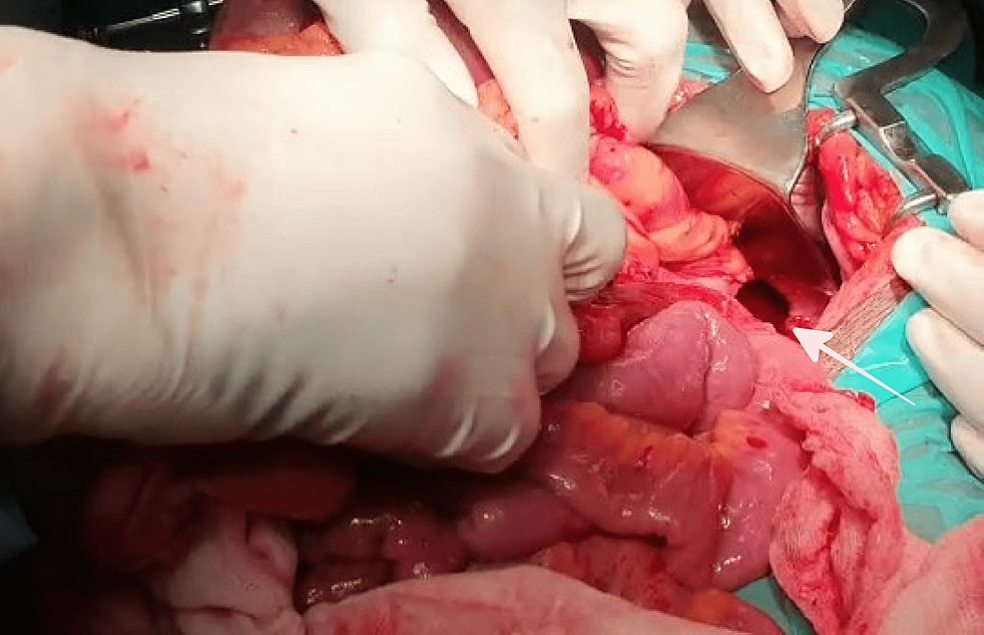
Intraoperative view of the left Bochdalek hernia defect (arrow) after reduction of herniated viscera.

Emergency LSCS with laparotomy was performed under general anesthesia, with the delivery of a live female baby of gestational age 28 weeks and birth weight 980 g. Intraoperatively, herniation of the stomach with organo-axial volvulus, distal transverse colon, splenic flexure, proximal descending colon, and spleen through left posterolateral diaphragmatic defect was noted (Figure 5).

**Figure 6:**
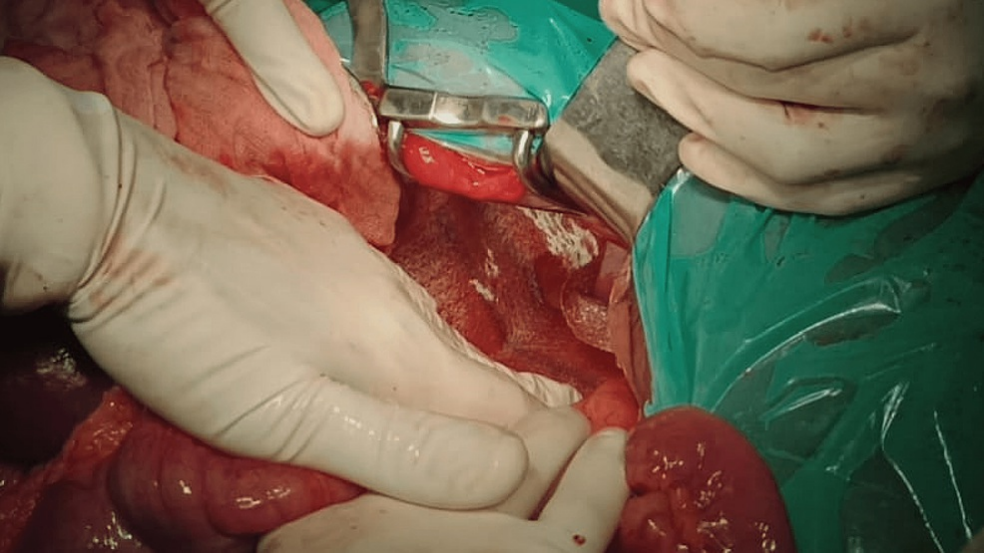
Placement of mesh after primary closure of the Bochdalek defect.

Herniated viscera were noted to be viable; the gastric volvulus and other contents were gently reduced after separating adhesions. An ectopic left kidney in the posterior thoracic wall was also identified and left in situ. The left-sided Bochdalek defect was primarily closed with 1-0 polypropylene and reinforced with a 10 cm × 15 cm composite dual mesh of oxidized regenerated cellulose (ORC) and polypropylene (Figure 6).

Postoperatively, the patient was kept on ventilator support for 24 hours. She was extubated on postoperative day 1 and transferred out of ICU on postoperative day 3. She made a full recovery and was discharged within two weeks, with minor respiratory complications. The neonate was preterm at 28 weeks with an Apgar score of 1 and 3 at 1 and 5 minutes, respectively, and required ventilator support and intensive care due to extreme prematurity and respiratory distress. The child expired in the neonatal ICU (NICU) after one day, despite best efforts.

**Figure 7:**
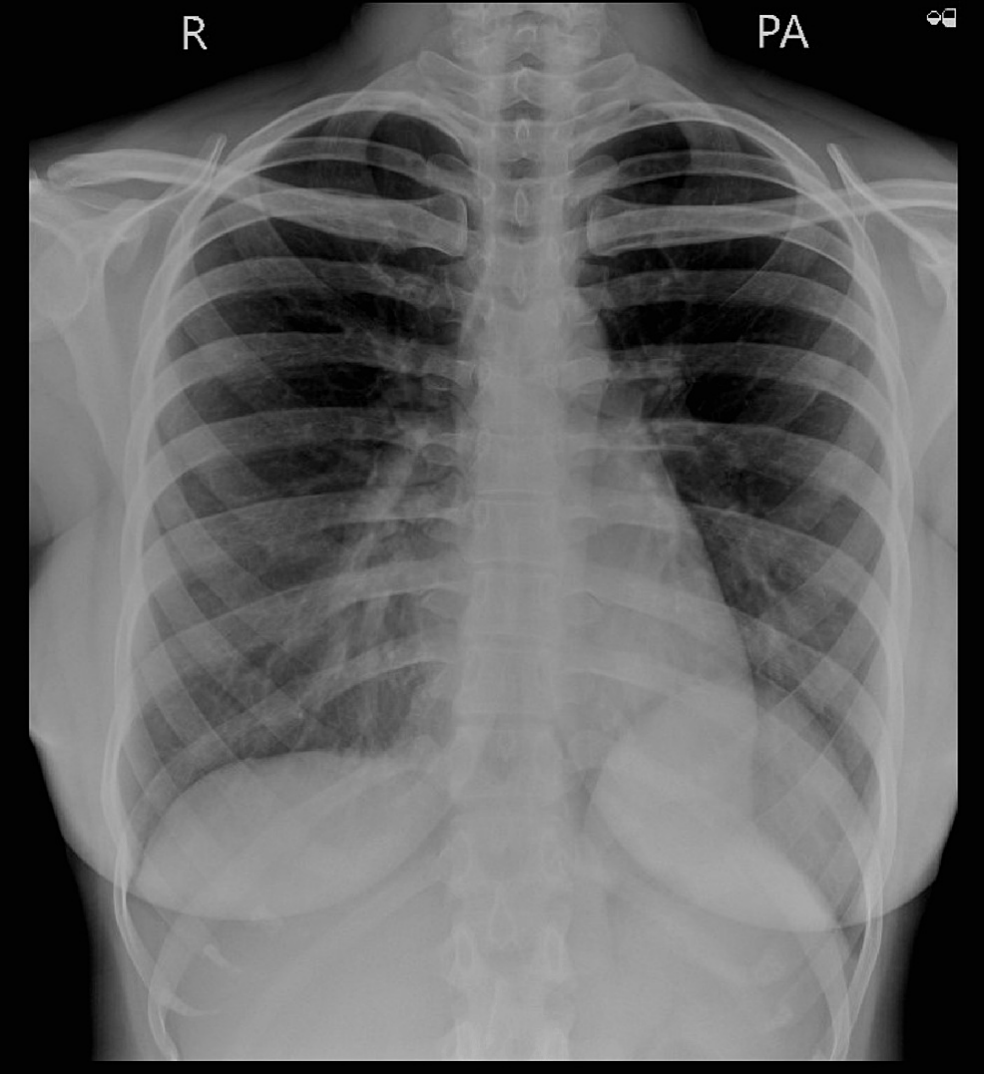
Chest radiograph PA view taken at the six-month follow-up. PA, posteroanterior

The patient reported no long-term sequelae on follow-up after one year. Her chest X-ray was normal at review (Figure 7). She was advised to wait a year before conceiving again, given the post-LSCS status.

## Discussion

Gastric volvulus is defined as the abnormal rotation of all or part of the stomach around one of its axes and can be categorized as primary (idiopathic) or secondary based on etiology [4,8]. They are known to occur in conjunction with abnormal gastric anatomy, laxity of gastric ligaments, diaphragmatic and para-esophageal hernias, diaphragmatic eventration, and phrenic nerve paralysis. Few cases of gastric volvulus secondary to diaphragmatic hernia presenting during pregnancy have been reported [9,10].

Pregnancy has often been cited as a precipitating factor in the delayed presentation of Bochdalek hernia and, as per a 2016 literature review, was reported in 5.3% of patients presenting with the same. Other recorded risk factors include congenital anomalies such as situs inversus, liver hypoplasia, ectopic kidney, and vascular anomalies [3]. The onset of symptoms during pregnancy in previously asymptomatic cases of CDH has been attributed to the increased intra-abdominal pressure caused by a progressively expanding gravid uterus, and progesterone-induced smooth muscle relaxation and stretching of the diaphragm, which renders it susceptible to elevation in intra-abdominal pressure [6,11].

Nevertheless, the actual incidence of symptomatic CDH during pregnancy is unknown and can occur regardless of maternal age and parity. Patients may be initially asymptomatic and develop symptoms during advanced gestational weeks as herniation occurs or even present postpartum [12]. The majority of cases diagnosed in pregnancy usually present in the second or third trimester as surgical emergencies, with a higher risk of complications in the third trimester [3,7]. In their 2021 review, Choi et al. reported preterm labor and fetal/neonatal loss in 35% and 16% of cases, respectively [7].

The major maternal complications are compression atelectasis of the lungs, respiratory failure, and strangulation of the herniated internal organs. The risk of maternal mortality increases with the development of gastrointestinal obstruction, possibly due to respiratory distress, bowel strangulation and perforation, fetal hypoxia, and acidosis [13]. In our patient, respiratory compromise and acute gastric volvulus led to rapid deterioration despite resuscitation, with the development of severe alkalosis and fetal distress. Choi et al. reported a maternal mortality rate of 11% in patients developing bowel obstruction, ischemia, or perforated viscera, due to sepsis and multisystem organ failure, despite surgical intervention [7].

Chest radiographs, ultrasound, CT, and magnetic resonance imaging (MRI) can be used for the diagnosis of Bochdalek hernias [2]. Chest radiographs can be used safely during pregnancy with a sensitivity of 70% [13]. Chest radiographical features include displacement of the mediastinum, air bubbles above the diaphragm level, and an opacified hemithorax. However, a normal view of a chest X-ray cannot exclude diaphragmatic herniation, and it can be difficult to diagnose a diaphragmatic hernia with only a chest radiographic examination [3,7].

Ultrasonography is widely used in obstetric imaging due to the absence of nonionizing radiation, easy accessibility, and real-time scanning. Ultrasonography features of CDH include a fragmented diaphragm, inability to identify the liver, spleen, kidney, the superior mesenteric and portal vessels within the normal position in the abdomen, displacement of the heart across the mediastinum, and the identification of bowel and liver in the chest; nevertheless, ultrasonography is insufficient for an adequate diagnosis [3,7].

In this patient, a diagnostic dilemma was posed by the previous sonological diagnosis of left diaphragmatic eventration and the detection of elevated lipase. CT evaluation confirmed left diaphragmatic hernia with herniation of splenic flexure, stomach, spleen, and pancreas, thus explaining the elevated lipase; the presence of concurrent acute gastric volvulus was also detected on CT.

CT remains the gold standard for diagnosing various types of diaphragmatic hernias, with a sensitivity and specificity of 78% to 82% and 87%, respectively [1,2]. As per the National Council on Radiation Protection and Measurements guidelines on radiation doses during pregnancy, exposure to ≤5 rad is considered negligible compared to the other risks of pregnancy. Since fetal radiation exposure from a single-acquisition abdominal/pelvis CT is 2.5 rad (25 mGy), the use of CT imaging is appropriate when imaging examinations based on the use of nonionizing radiation fail to yield the necessary clinical information and if the benefits of diagnosis exceed the theoretical risk of fetal exposure [14]. MRI may be applicable in pregnant patients who are hemodynamically stable [2].

Recommendations regarding the management of diaphragmatic hernia in pregnant patients are based on clinical presentation and gestational age [6]. If diagnosed in the first or second trimester, surgical repair (laparoscopic or open) may be performed during the second trimester once organogenesis is achieved. In the absence of symptoms, third-trimester patients can be managed expectantly till fetal maturity is attained, after which Caesarean section delivery and hernia repair may be undertaken [7]. The development of complications such as obstruction, strangulation, or respiratory compromise requires immediate surgical treatment.

Following the repair of the diaphragmatic hernia, vaginal delivery (without bearing down) may be safely conducted as uterine contractions do not increase the intraabdominal pressure and are unlikely to cause rupture at the repaired site [11,15]. Repaired diaphragmatic hernia is not a contraindication for future pregnancies.

## Conclusions

In conclusion, diaphragmatic hernia should be ruled out with CT in women of reproductive age diagnosed with diaphragmatic eventration, as ultrasonography is insufficient for an adequate diagnosis. If detected during pregnancy, an elective repair can be offered in the second trimester to prevent antenatal and peripartum complications. The onset of obstructive features in otherwise expectantly managed patients is a surgical emergency irrespective of gestational age and fetal maturity.
